# Integrative Physiological, Transcriptional, and Metabolic Analyses Provide Insights Into Response Mechanisms of *Prunus persica* to Autotoxicity Stress

**DOI:** 10.3389/fpls.2021.794881

**Published:** 2021-12-15

**Authors:** Wanqi Shen, Chunfa Zeng, He Zhang, Kaijie Zhu, Hao He, Wei Zhu, Hanzi He, Guohuai Li, Junwei Liu

**Affiliations:** ^1^Key Laboratory of Horticultural Plant Biology, Ministry of Education, College of Horticulture and Forestry Sciences, Huazhong Agricultural University, Wuhan, China; ^2^Haikou Experimental Station, Chinese Academy of Tropical Agricultural Sciences, Haikou, China; ^3^College of Life and Environmental Sciences, Hunan University of Arts and Science, Changde, China; ^4^Institute of Fruit and Tea, Hubei Academy of Agricultural Sciences, Wuhan, China; ^5^College of Plant Science and Technology, Huazhong Agricultural University, Wuhan, China

**Keywords:** plant autotoxicity, benzoic acid, *Prunus persica*, replant problem, stress response

## Abstract

Autotoxicity is known as a critical factor in replanting problem that reduces land utilization and creates economic losses. Benzoic acid (BA) is identified as a major autotoxin in peach replant problem, and causes stunted seedling growth or even death. However, the physiological and molecular mechanisms of peach response to BA stress remain elusive. Here, we comprehensively studied the morphophysiological, transcriptional, and metabolic responses of peach plants to BA toxicity. Results showed that BA stress inhibited peach seedlings growth, decreased chlorophyll contents and fluorescence levels, as well as disturbed mineral metabolism. The contents of hydrogen peroxide, superoxide anion, and malondialdehyde, as well as the total antioxidant capacity, were significantly increased under BA stress. A total of 6,319 differentially expressed genes (DEGs) were identified after BA stress, of which the DEGs related to photosynthesis, redox, and ion metabolism were greatly changed; meanwhile, numerous stress-responsive genes (*HSPs*, *GSTs*, *GR*, and *ABC transporters*) and transcription factors (*MYB*, *AP2/ERF*, *NAC*, *bHLH*, and *WRKY*) were noticeably altered under BA stress. BA induced metabolic reprogramming, and 74 differentially accumulated metabolites, including amino acids and derivatives, fatty acids, organic acids, sugars, and sugar alcohols, were identified in BA-stressed roots. Furthermore, an integrated analysis of genes and metabolites indicated that most of the co-mapped KEGG pathways were enriched in amino acid and carbohydrate metabolism, which implied a disturbed carbon and nitrogen metabolism after BA stress. The findings would be insightful in elucidating the mechanisms of plant response to autotoxicity stress, and help guide crops in alleviating replant problem.

## Introduction

Peach (*Prunus persica*) is an economically important fruit crop worldwide, with a global yield of approximately 25 million tons annually, of which above half are produced in China (Zheng et al., 2021). With the increased demand and the reduced cultivated land acreage of peach, replant problem (also known as replant disease) has become increasingly prominent (He et al., 2019). Replant problem refers to a phenomenon of the successive plantation of same or similar plant species on the same land, resulting in reductions of crop yield and quality under usual cultivation practices (Zhu et al., 2017; Yin et al., 2018). Of the stone fruits, peach replant problem is much more serious than other species within the *Prunus* genus owing to the shorter life cycle of peach trees and fast replacement of new cultivars, and has become an important factor hindering the sustainable development of peach industry (He et al., 2019).

Replant problem affects a wide variety of plant species worldwide and causes severe losses in forestry and agriculture (Dong et al., 2018). The underlying factors for replanting problems are largely attributed to allelopathic autotoxicity, microbial community shift, and soil fertility imbalance (Guo et al., 2016; Yim et al., 2020; Zhang et al., 2020c). For example, the reduction of rhizosphere microbial diversity is one of the major factors in peanut monocropping (Li et al., 2019). The abundances of *Fusarium* and *Mortierella* species in the soil are significantly correlated with apple replant disease severity (Li et al., 2021). In addition to biotic factors, poor soil nutrition or structure could also lead to the occurrence of replant problems (Gao et al., 2020). For example, soil nutrient availability and soil enzyme activities are comparatively lower under monocropping conditions than in the intercropping system (Xiao et al., 2019). In peach replanted soil, the contents of available N, P, and K are significantly decreased compared with non-replanted soil (He et al., 2019). The dilemma of soil fertility and pathogenic microorganisms could be greatly solved by nutrient restoration and soil disinfection (He et al., 2019; Nyoni et al., 2019). In contrast, autotoxicity is a knotty problem due to the complicated generation, accumulation, and detoxification mechanisms of various autotoxins.

Autotoxicity is a special kind of allelopathy, and is considered to be a major factor resulting in the prevalence of replanting problem (Xie et al., 2017; Lobón et al., 2019; Sun and He, 2019). Autotoxins are released into the surrounding environment through leaching, volatilization, root secretion, and residue decomposition, and have a pronounced growth inhibition on the same or similar plant species (Liu et al., 2019a; Gallego et al., 2020; Zhang et al., 2020b). Compounds such as phenolic compounds (phenolics, flavonoids, coumarins, and quinones), terpenoids (mono-, sesqui-, di-, tri-terpenes, and steroids), alkaloids, and nitrogen-containing chemicals (non-protein amino acids, benzoxazinoids, and cyanogenic glycosides) have been suggested to be the primary autotoxins (Cheng and Cheng, 2015; Kong et al., 2019).

Autotoxins interfere in plant growth and developmental processes at multiple levels. They work fundamentally in two facets: (1) act as chemical signals in the interaction between plants and microorganisms that can change the microbial population structure thus indirectly affect plant growth. For example, vanillic acid alters soil fungal community structure, principally by modifying the composition and diversity of *Fusarium* spp. and *Trichoderma* spp. in the cucumber rhizosphere (Chen et al., 2018). Coumarins could selectively repress the soil-borne fungal pathogens, and induce rhizobacteria production to promote plant health (Stringlis et al., 2018). High levels of soil BA play a crucial role in forming the microbial composition of peach replanted soil (He et al., 2021); (2) work as a stress factor that directly disturbs plant functioning. For instance, phytotoxin (-)-catechin is secreted from *Centaurea maculosa* could inhibit the growth and germination of native species in field soil at natural concentration (Bais et al., 2003). The rhizosphere soil extracts of *Astragalus* plants inhibit their own seedling growth (Guo et al., 2016). Cyclic dipeptide is a highly active phytotoxin that restricts offspring growth in the replanted Chinese fir tree plantations (Chen et al., 2014). Moreover, autotoxins could also modify plant root secretion characteristics, such as *p*-hydroxybenzoic acid induces the exudation of salicylic acid from the root border cells of grapevine (Liu et al., 2019a,b). Approximately 5–30% of the net carbon fixed during photosynthesis is released into the rhizosphere through root exudation (Chen et al., 2014; Kong et al., 2019). Autotoxins as a kind of important root exudates drive plant-soil feedbacks, thus the specific responsive mechanisms of plants to autotoxins are worthy of in-depth exploration.

Benzoic acid (BA) is a well-known autotoxin and identified from root exudates and rhizosphere soils of various plant species, which causes severe allelopathic suppression (Asaduzzaman and Asao, 2012; Zhang et al., 2018). BA also has been widely detected in peach roots and replanted soils, and exhibits dramatic inhibition on peach plant growth (Zhu et al., 2017). The previous studies mostly focus on morphological changes and mitigation strategies under BA stress, but the information of transcriptional and metabolic adaptations of plants to BA stress is quite limited (Zhu et al., 2017; He et al., 2019). In this study, the morphophysiological changes of peach plants under BA stress were first investigated using hydroponic systems. Further, the molecular mechanisms of peach plants response to BA stress were explored using an integrated omics approach. The findings will shed new insights into the specific response mechanisms of peach under autotoxicity stress, and provide valuable information for developing replant-tolerant materials of peach.

## Materials and Methods

### Plant Growth Conditions and Stress Treatments

Seeds of peach [*P. persica* (L.) Batsch] rootstock were stratified in moist sand (4°C, 12 weeks) to sprout, and then transplanted to 32-hole trays filled with media containing nutrient soil (Xingyuxing, China), peat moss (Pindstrup, Denmark), and vermiculite (Taoyang, China) at 3:1:1 (*v*:*v*:*v*). The morphologically uniform 10-d-old seedlings were randomly selected and transferred to plastic tubs (28.5 cm × 19.5 cm × 7.0 cm) containing 2 L half-strength Hoagland nutrient solution. After 5 days pre-culture, the seedlings were exposed to nutrient solution supplemented with 0, 0.2, 0.4, and 0.8 mM BA (Sinopharm, China). All treatments were performed with three independent biological replicates, and each replicate consisted of 4 individual plantlets and arranged in one tub. All tubs were wrapped with black tapes to limit light exposure and placed in a growth chamber at 25°C, 65% relative humidity with 16 h of light (300 μmol m^–2^ s^–1^). The nutrient solution was adjusted to pH 5.8 with KOH, and refreshed every 3 days. The solution was continuously aerated with an air pump in each tub, and dissolved oxygen concentration was maintained at 8.0–8.5 mg L^–1^ by an automatic monitor (Leici, China).

### Morphological, Physiological, and Biochemical Assays

The performance of peach seedlings was photographed before and after the BA treatment using a high-resolution camera (Canon, Japan). Plant height (from the stem base to the terminal bud) was recorded at 0 (initial) and 120 h (final) from the onset of BA treatment. At harvest, the whole plants were divided into aboveground parts (shoots and leaves) and roots, and their fresh biomass was separately weighed in every BA treatment group. Root architecture was scanned and analyzed using the WinRHIZO^®^ image analysis system (v2019a, Canada).

For chlorophyll fluorescence analysis, peach plants were placed in darkness for 30 min prior to measuring with an IMAGING-PAM chlorophyll fluorimeter (Walz, Effeltrich, Germany), based on which maximum quantum efficiency of photosystem II (*Fv*/*Fm*) was calculated using Imaging WinGegE software. Chlorophyll was extracted and assayed as reported previously (Shen et al., 2021). Briefly, 0.1 g of freshly harvested leaf samples were incubated in 10 mL of 80% acetone (*v*/*v*), and the chlorophyll (a, b, and total) contents were analyzed with a spectrophotometer (Shimadzu, Japan). Root vigor was determined by the triphenyl tetrazolium chloride (TTC) method and expressed as the capacity for deoxidization (mg g^–1^ FW h^–1^). The contents of malondialdehyde (MDA), hydrogen peroxide (H_2_O_2_), and the activity of anti-superoxide anion (anti-O_2_^•–^; negatively proportional to O_2_^•–^ levels) were examined using the appropriate detection kits (A003-3-1, MDA; A064-1-1, H_2_O_2_; and A052-1-1, anti-O_2_^•–^) from Nanjing Jiancheng Bioengineering Institute (China) following the manufacturer’s manuals. Total antioxidant capacity (T-AOC) was measured using an assay kit (ABTS-1-D, Suzhou Keming Biotechnology, China) according to the manufacturer’s instructions.

The oven-dried leaves and roots were finely ground separately for nutrient elemental analysis. For each sample, 0.1 g of tissue samples were homogenized and digested with nitric acid (AR, 65%; Sinopharm, China). Inductively coupled plasma mass spectrometry (ICP-MS; Thermo Fisher Scientific, United States) was used to measure the contents of mineral elements (Shen et al., 2021).

### Transcriptome Sequencing and Quantitative Real-Time PCR Analysis

Total RNA samples from 0.4 mM BA treated-peach roots at six time points (0, 6, 12, 24, 72, and 120 h) were extracted using TRIzol^®^ Reagent (Invitrogen, United States) according to the manufacturer’s instructions. A total of 18 libraries (one treatment × six time points × three replicates) were constructed and sequenced using Illumina HiSeq X ten/NovaSeq 6000 system (2 × 150 bp read length) at Majorbio Biological Technology (Shanghai, China). After removing adapters and discarding low-quality sequences, the clean reads were aligned to the reference genome of *P. persica* (v2.0.a1) using HISAT2 (v2.1.0) and Bowtie2 (v2.4.1) software. The expression level of each transcript was calculated as the number of fragments per kilobase per million mapped reads (FRKM) using RSEM (v1.3.1) software. DESeq R package (v1.24.0) was used to identify differentially expressed genes (DEGs) between different treatment groups. Genes with false discovery ratio < 0.05 and | log_2_ (fold change)| ≥ 1 were considered as significantly DEGs. Diamond software (v0.9.24) was used to annotate the functional information of NR (NCBI non-redundant protein sequences), KOG (Eukaryotic orthologous groups), Swiss-Prot (Swiss-Prot protein sequence database), Gene Ontology (GO), and Kyoto Encyclopedia of Genes and Genomes (KEGG). Enrichment analysis was analyzed using the KEGG database to obtain a detailed description of the DEGs.

One microgram of extracted RNA was reverse-transcribed into single-strand DNA using HiScript II Q RT superMix for qRT-PCR (Vazyme, China) following the manufacturer’s manuals. Several randomly selected genes were subjected to qRT-PCR analysis to validate the reliability of RNA-seq data. The gene specific primers were designed using Primer3Plus and synthesized by Tsingke Biological Technology (Wuhan, China). Primer sequences for qRT-PCR analysis are presented in Supplementary Table 1. The qRT-PCR reactions were set up in 10 μL volumes using Hieff™ qPCR SYBR Green Master Mix (Yeasen, China) on a QuantStudio 6 Flex system (Applied Biosystems, United States). Relative expression levels were normalized with *translation elongation factor 2* (*TEF2*) gene of *P. persica* according to the comparative 2^−ΔΔCT^ method (Zhang et al., 2020a; Shen et al., 2021).

### Metabolite Profiling

The root samples harvested at 0 (control), 72, and 120 h after BA treatments (each time point had six biological replicates and 8 plantlets per replicate) were sent for non-targeted gas chromatography-mass spectrometry (GC-MS) analysis. The metabolites were extracted from 50 mg samples using 500 μL of 80% methanol (*v*/*v*) containing 0.3 mg mL^–1^ internal standard 2-chloro-L-phenylalanine (Sigma, United States). The sample mixtures were homogenized at 50 Hz for 3 min, and sonicated in ice water for 10 min, then placed at −20°C for 30 min. After centrifugation at 5,000 × *g* at 4°C for 20 min, the supernatants were transferred to glass vials and dried with speed vacuum. The extracted compounds were derivatized using N,O-bis(trimethylsilyl)trifluoroacetamide (Sigma, United States), then analyzed by an Agilent 8890B gas chromatography system coupled to an Agilent 5977B mass selective detector (Agilent Technologies, United States). A HP-5MS capillary column (30 m × 0.25 mm × 0.25 μm; Agilent J&W Scientific, United States) was employed for metabolite separation. Helium (>99.999%) acted as carrier gas at a constant flow rate of 1 mL min^–1^ through the column. The injector temperature was maintained at 260°C, and the injection volume was 1 μL in splitless mode. The temperature of ion source and MS quadrupole were set at 230°C and 150°C, respectively. The initial oven temperature (60°C) was constantly increased to a final temperature of 310°C by 8°C min^–1^. The mass spectra were recorded at 3.2 scans s^–1^ with an m/z 50–500 scanning range after a solvent delay of 5 min.

The raw data were processed with MassHunter Workstation Quantitative Analysis software (v10.0.707.0; Agilent Technologies, United States) to obtain the three-dimensional data matrix, which contained sample information, metabolite name, and mass response intensity. A multivariate statistical analysis was performed using R packages (ropls, v1.6.2). All samples were tested to visualize the metabolic alterations by principal component analysis (PCA). Metabolites with *P-*value of *t*-test < 0.05 and variable influence on projection (VIP) values of ≥1 were considered differentially accumulated metabolites (DAMs). The metabolic enrichment pathways were constructed using the KEGG metabolic database.

### Statistical Analysis

Benzoic acid treatments in this study were biologically repeated at least three times. All data represent mean values ± SE of replicates. Different letters above the bars indicate statistically significant difference at *P* < 0.05 as obtained by One-way analysis of variance (ANOVA) based on Tukey’s *post hoc* test of GraphPad Prism version 8.0.2 (GraphPad Software, Inc., La Jolla, CA, United States)^1^. Asterisks denote Student’s *t-*test significance: *P* < 0.05 (*). The transcriptomic and metabolic data were analyzed on Majorbio Cloud Platform^2^.

## Results

### Effects of Exogenous Benzoic Acid Treatments on Plant Morphology

**FIGURE 1:**
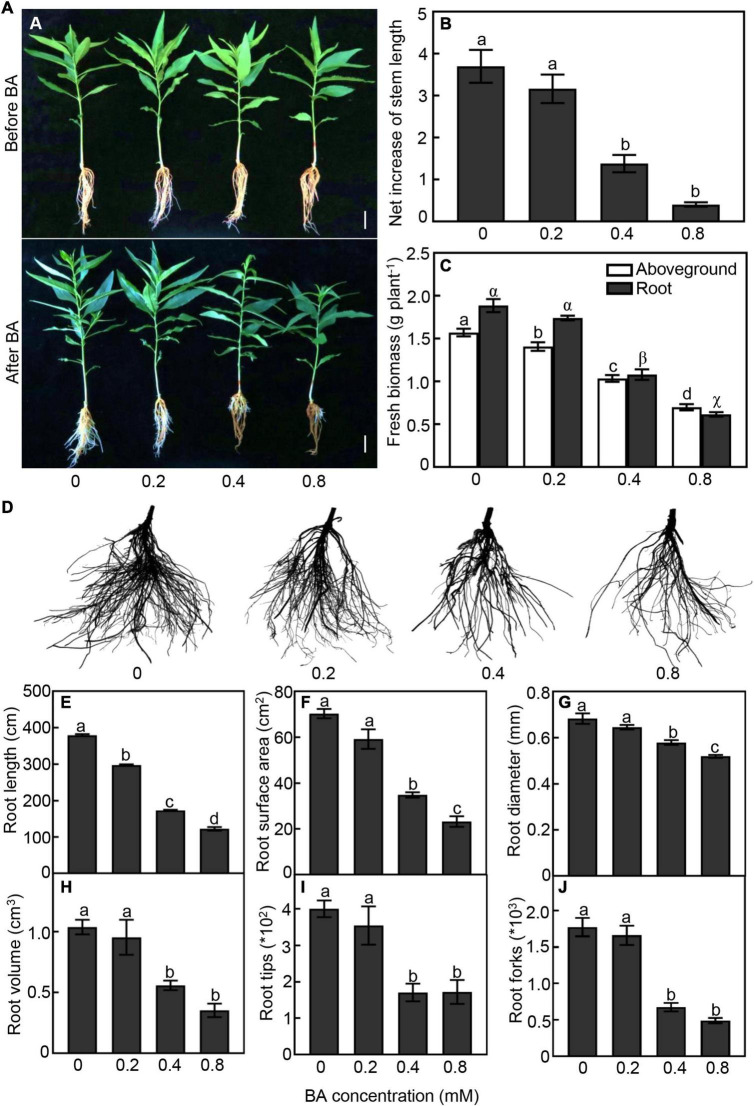
Growth performan ce of peach seedlings in response to different concentrations of benzoic acid (BA) treatment for 120 h. **(A)** Morphological change of peach plants before and after BA treatment. Bar represents 2 cm. **(B)** Net increase of stem length was calculated by the height at 120 h after BA treatment and the height at the onset of BA treatment. **(C)** Aboveground and root fresh biomass. **(D)** Morphological change of root system. **(E)** Total root length. **(F)** Root surface area. **(G)** Average of root diameter. **(H)** Root volume. **(I)** Number of root tips. **(J)** Number of root forks. Error bars indicate ± SE. Different letters on top of bars indicate significant difference between BA treatments at *P* < 0.05.

We first tested different concentrations of BA (0, 0.2, 0.4, and 0.8 mM) on the growth performance of peach seedlings. As expected, peach morphology behaved differently to various BA treatments. Root system of peach seedlings gradually became brown with the increase of BA concentration (Figure 1A). Higher concentrations of BA showed strong inhibition on seedlings growth. In the 0.4 and 0.8 mM BA treatments, the net increase of stem length was significantly reduced by 63 and 89%, and the fresh biomass of aboveground part was decreased by 34 and 56%, respectively, when compared with the control (Figures 1B,C).

The belowground part was also strongly impacted by the BA treatments (Figures 1C–J). Under 0.4 and 0.8 mM treatments, the root biomass was significantly reduced by 43 and 65%, respectively, compared with the control (Figure 1C). With the increase of BA concentration, the damage to the root system was aggravated obviously (Figure 1D). Total root length of the 0.2, 0.4, and 0.8 mM BA treated seedlings was 21, 59, and 71% less than that of the control, respectively (Figure 1E). Root surface area of the 0.4 and 0.8 mM BA-treated was significantly reduced by 55 and 63%, respectively (Figure 1F). Moreover, the root average diameter, volume, and tip and fork numbers in the 0.4 mM BA treatment were significantly reduced by 40, 37, 57, and 62%, respectively, compared to the control (Figures 1G–J). Collectively, the results indicated that BA treatments inhibited plant growth and root development, and a significant inhibition starting at 0.4 mM BA.

### Physiological and Biochemical Changes to Benzoic Acid Treatment

**FIGURE 2:**
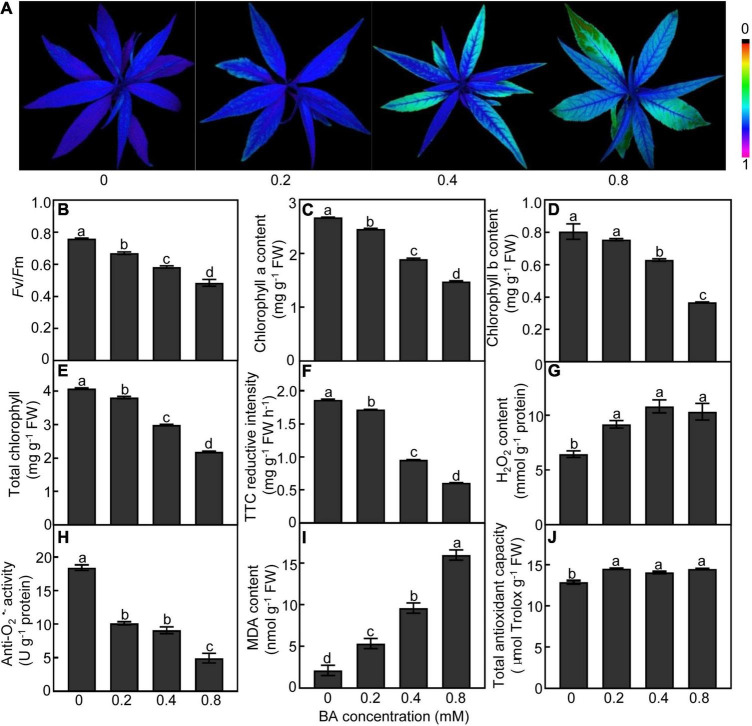
Physiological response of peach plants after BA treated for 120 h. **(A)** Chlorophyll fluorescence imaging. **(B)** Maximum potential of PSII efficiency (*F*v/*F*m). **(C)** Chlorophyll a content. **(D)** Chlorophyll b content. **(E)** Total chlorophyll content. **(F)** Root vigor. **(G)** H_2_O_2_ content. **(H)** Anti-O_2_^•–^ activity (a parameter negatively proportional to O_2_^•–^ level). **(I)** MDA content. **(J)** Total antioxidant capacity. Data are means ± SE (*n* = 3). Different letters on top of bars denote significant differences between treatments at *P* < 0.05.

Exogenous BA supply caused dramatic changes in the photosynthetic capacity of peach leaves (Figures 2A–D). The leaves treated with BA at higher concentrations (0.4 and 0.8 mM) showed lower chlorophyll fluorescence than the control (Figure 2A). Both *Fv/Fm* ratios and chlorophyll (a, b, and total) contents were consistently and continually decreased with the increase of BA dosage (Figures 2B–E). The root vigor was decreased by 49 and 68% in the 0.4 and 0.8 mM BA-treated groups, respectively, compared to the control (Figure 2F). The contents of H_2_O_2_ in roots were significantly increased after BA treatment (Figure 2G); meanwhile, the activities of anti-O_2_^•–^, which negatively correlates with O_2_^•–^ levels, were significantly decreased by 50 and 73%, respectively, in the 0.4 and 0.8 mM BA-treated roots as compared with the control (Figure 2H). The MDA contents were 1. 5-, 3. 5-, and 6.5-fold higher in the 0.2, 0.4, and 0.8 mM BA-treated than the control, respectively (Figure 2I). Total antioxidant capacity, which includes both the enzymatic and non-enzymatic systems, was significantly increased upon BA treatments, but showed no significant difference between BA treatments (Figure 2J).

The mineral elements were measured in roots and leaves of peach plants treated with 0.4 mM BA or not for 120 h. In terms of macro-elements, a significant decrease of P content was observed in BA-treated leaves (44%) and roots (24%) compared to the control. The K content in the leaves and roots under BA treatment was significantly reduced by 59 and 32%, respectively. In roots and leaves, the amount of Mg was lowered by 41 and 15%, respectively, when compared with the control (Table 1). The content of Ca in the BA-imposed roots was significantly increased by 69%, whereas decreased by 12% in the leaves compared to the control, suggesting BA might affect Ca transport (Table 1). For the trace elements, the contents of Mn and Zn in roots were significantly decreased by 63 and 49%, respectively, compared to the control. In addition, the levels of Fe and B were significantly increased in roots, but decreased in leaves. The content of Cu was not affected by BA treatment (Table 1). The results indicated that BA treatment markedly interrupted the mineral metabolism of both peach leaves and roots.

**TABLE 1:** Effects of BA stress on mineral contents in roots and leaves of peach seedlings.

Element	Tissue	Control	BA
P (mg g^–1^ DW)	Roots	12.79 ± 0.20	7.18 ± 0.08*
	Leaves	7.58 ± 0.01	5.73 ± 0.06*
K (mg g^–1^ DW)	Roots	44.76 ± 1.00	18.59 ± 0.21*
	Leaves	32.47 ± 0.67	21.77 ± 0.27*
Ca (mg g^–1^ DW)	Roots	17.93 ± 0.28	30.38 ± 0.45*
	Leaves	7.83 ± 0.05	6.90 ± 0.08*
Mg (mg g^–1^ DW)	Roots	3.50 ± 0.05	2.06 ± 0.02*
	Leaves	5.58 ± 0.01	4.75 ± 0.03*
Fe (μg g^–1^ DW)	Roots	384.36 ± 4.79	496.44 ± 5.63*
	Leaves	154.10 ± 1.62	133.41 ± 1.90*
Mn (μg g^–1^ DW)	Roots	124.82 ± 2.09	45.97 ± 0.40**
	Leaves	69.53 ± 0.09	65.70 ± 0.65*
Cu (μg g^–1^ DW)	Roots	20.09 ± 1.01	19.08 ± 0.43
	Leaves	12.87 ± 0.44	12.95 ± 0.38
Zn (μg g^–1^ DW)	Roots	58.88 ± 0.86	29.83 ± 0.43*
	Leaves	45.66 ± 2.98	35.71 ± 0.66*
B (μg g^–1^ DW)	Roots	20.06 ± 0.14	21.75 ± 0.14*
	Leaves	75.23 ± 0.38	60.92 ± 1.14*

*Control, half-strength Hoagland nutrient solution; BA, half-strength Hoagland nutrient solution supplemented with 0.4 mM BA. Data are means ± SE (n = 3). Asterisks in column represent the statistical significance between the control and BA-treated groups at P < 0.05.*

### Transcriptomic Response of Peach Roots to Benzoic Acid Treatment

**FIGURE 3:**
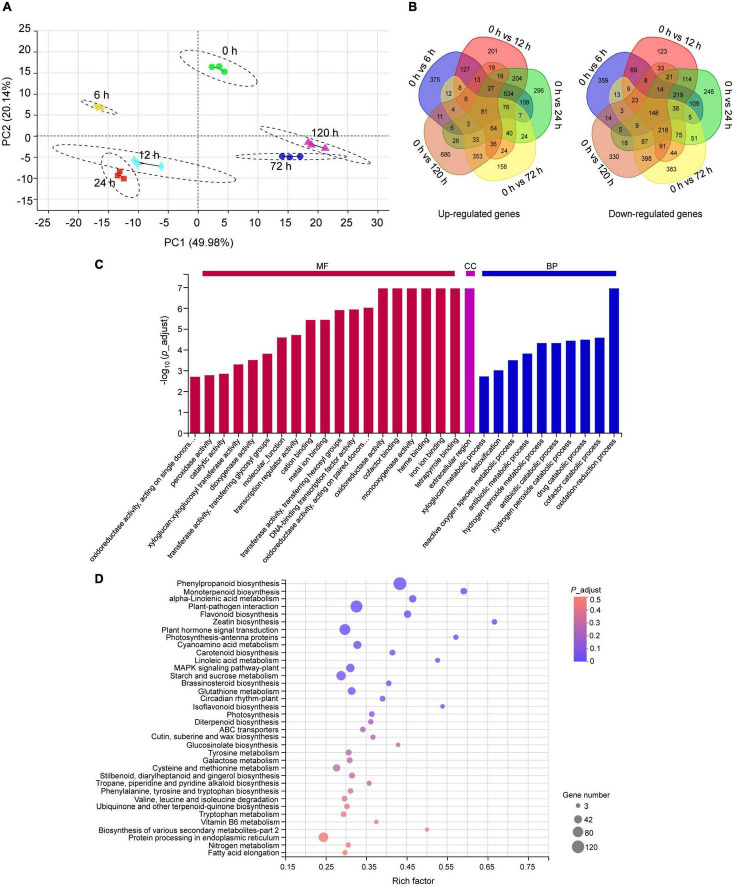
Transcriptome analysis of peach roots treated with 0.4 mM BA. **(A)** Principal component analysis (PCA) of transcriptomic data. **(B)** Venn diagram display of all upregulated and downregulated differentially expressed genes (DEGs) across different time intervals. **(C)** Summary of Gene Ontology (GO) categories of the DEGs. MF, molecular function; CC, cellular component; BP, biological process. **(D)** The Kyoto Encyclopedia of Genes and Genomes (KEGG) pathway enrichment scatter map. The bubble size represents the number of DEGs detected in the corresponding pathway. The larger bubble diameter means more DEGs annotated to this pathway. The rich factor is the ratio of DEGs to the total background gene number in each pathway. The color of the bubble changed from red to purple, indicating that the *P-*adjust gradually decreases. When *P*-adjust < 0.05, the pathway is defined as a significantly enriched one.

To understand the molecular mechanism underlying peach plants response to BA stress, an RNA-seq was performed on samples from 0.4 mM BA treated roots at 0 (control), 6, 12, 24, 72, and 120 h. After filtering low-quality reads and adapters, we obtained an average of 44.97–50.98 million clean reads. More than 94% of the clean data had scores greater than Q30 in each library. In total, 92 to 95% of clean reads were mapped to the peach reference genome (Supplementary Table 2). A total of 25,763 genes were detected and the expression levels Log_10_ (FPKM) of detected genes in all samples ranged from −1.7 to 4.5 (Supplementary Figure 1A). For functional annotation, the unigenes showed high alignment to NR (99%) and COG (91%) databases (Supplementary Figure 1B). Pearson correlation coefficient analysis indicated a high precision between replicates (*R*^2^ > 0.99) (Supplementary Figure 1C). In principal component analysis, samples were clearly separated, and principal component 1 and 2 (PC1 and PC2) explained 70% of the total changes (Figure 3A). A total of 6,319 DEGs were identified after BA stress, of which 2,443, 2,726, 2,925, 2,524, and 2,807 DEGs were detected at 6, 12, 24, 72, and 120 h compared to control, respectively (Figure 3B).

The functions of all DEGs were classified using the GO database. Within the molecular function category, the DEGs were abundantly enriched in “tetrapyrrole binding,” “iron ion binding,” “heme binding,” “monooxygenase activity,” “cofactor binding,” and “oxidoreductase activity.” In the biological processes, the DEGs were mainly enriched in “oxidation-reduction process” and “cofactor catabolic process.” A category “extracellular region” was dominant in the cellular component (Figure 3C). Furthermore, the KEGG pathway enrichment analysis indicated that the identified 6,319 DEGs were enriched into 123 pathways, of which 12 pathways showing *P*-adjust < 0.05 (Supplementary Table 3). We found 429 DEGs were highly enriched in categories: “phenylpropanoid biosynthesis” (120), “monoterpenoid biosynthesis” (26), “α-linolenic acid metabolism” (33), “plant-pathogen interaction” (102), “flavonoid biosynthesis” (33), “glutathione metabolism” (38), “ABC transporters” (14), and “protein processing in endoplasmic reticulum” (63) (Figure 3D).

To validate the RNA-seq data, 12 DEGs related to glutathione metabolism, α-linolenic acid metabolism, phenylpropanoid biosynthesis, protein processing in endoplasmic reticulum, transcriptional regulation, and stress defense were selected for qRT-PCR. As expected, the trends of the resulting changes in the expression of the selected genes obtained by qRT-PCR were highly consistent with those of the RNA-seq data (*R*^2^ = 0.8501) (Supplementary Figure 1D), reflecting the RNA-seq data was reliable.

### Analysis of Differentially Expressed Genes Associated With Photosynthesis, Redox, and Ion Metabolism Pathways

Given that the BA stress greatly interfered with photosynthetic parameters, reactive oxygen species (ROS) balance, and ion metabolism of peach plants (Figure 2 and Table 1), we accordingly profiled the transcriptomic data corresponding to these pathways. Numerous photosynthesis-related genes were significantly downregulated by BA stress, including *serine/threonine-protein kinase* (*STPK*), *chlorophyll-binding protein* (*CBP*), *chlorophyll a oxygenase* (*CAO*), and *ferredoxin* (*FDX*) (Supplementary Table 4). BA stress dramatically increased the *respiratory burst oxidase homologs* (*RBOHs*) genes expression and significantly decreased the ROS scavenging-related genes expression, including *peroxidase* (*POD*), *catalase* (*CAT*), *superoxide dismutase* (*SOD*), and *ascorbate peroxidase* (*APX*) (Supplementary Table 4). Additionally, we observed 16 DEGs related to ion uptake or transport, including *potassium transporter* (*KT*), *ion channel proteins* (*ICP*), and *zinc transporter* (*ZnT*), of which 4 DEGs were upregulated and 12 DEGs were downregulated (Supplementary Table 4).

### Analysis of Differentially Expressed Genes Involved in Stress Response

**FIGURE 4:**
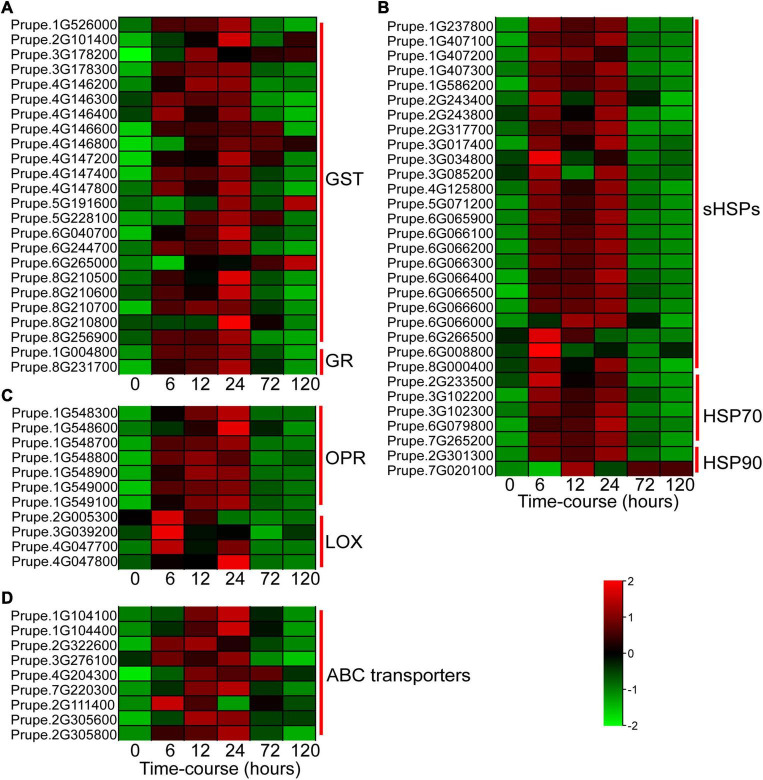
Heatmap showing the expression profiles of DEGs involved in BA stress response. **(A)** DEGs involved in glutathione metabolism. GST, glutathione S-transferases; GR, glutathione reductase. **(B)** DEGs related to protein processing in endoplasmic reticulum. sHSPs, small heat shock proteins; HSP, heat shock protein. **(C)** DEGs associated with α-linolenic acid metabolism. OPR, 12-oxophytodienoate reductase; LOX, lipoxygenase. **(D)** DEGs encoding ABC transporters. ABC transporters, ATP-binding cassette transporters. The colors of heatmap cells indicate scaled gene expression levels across different samples. The color from green to red indicates that the levels of gene expression gradually increase.

In this study, a great number of DEGs associated with stress response were significantly regulated by BA stress. Glutathione S-transferases (GSTs) catalyze the conjugation of glutathione to a wide variety of cytotoxic substrates to reduce their toxicity (Wang et al., 2020). BA stress sharply and significantly induced the expression levels of *GST* and glutathione reductase (*GR*) genes (Figure 4A). Twenty-four small heat shock proteins (*sHSPs*), five *HSP70*, and two *HSP90* were significantly upregulated at the early stage (6–24 h) of BA treatment (Figure 4B). Moreover, genes involved in α-linolenic acid metabolism, such as 12-oxophytodienoate reductase (*OPR)* and lipoxygenase (*LOX*), were dramatically induced by BA treatment (Figure 4C). Nine ABC transporters were also markedly upregulated within 6–24 h after BA treatment (Figure 4D). Interestingly, most of these stress-responsive genes expression showed a trend of upward and then downward, suggesting that they play important role in peach response to BA stress at the early stage.

### Identification of Transcription Factors Involved in Benzoic Acid Stress

**FIGURE 5:**
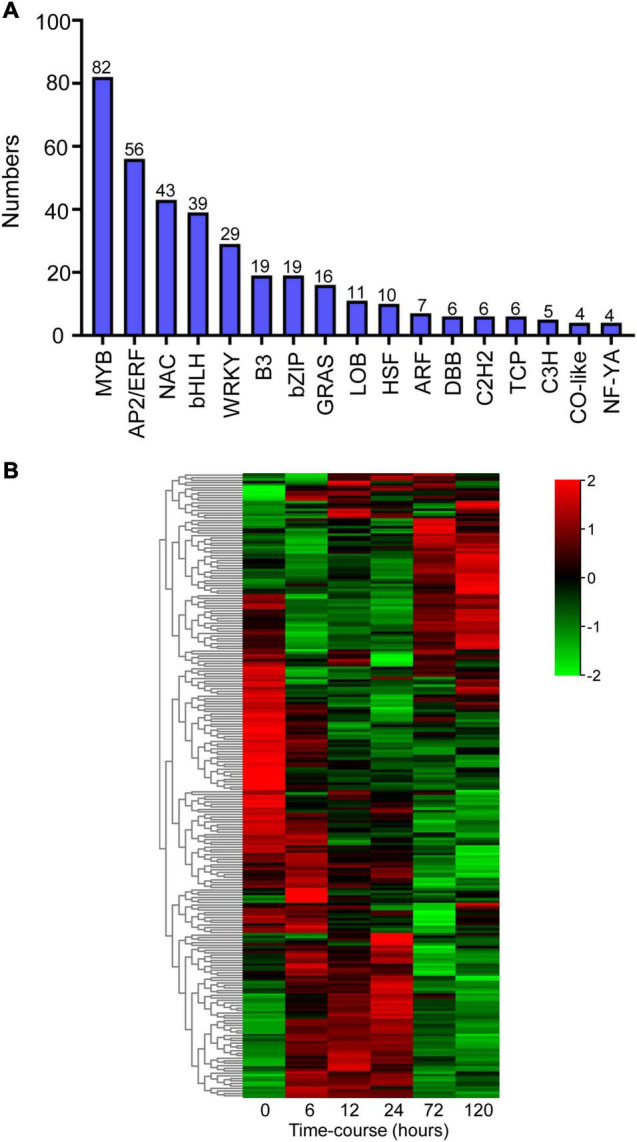
Identification of differentially expressed transcription factors (TFs) after BA stress. **(A)** Numbers of predicted TFs belongs to different families. **(B)** Hierarchical cluster heatmap of the common TFs (MYB, AP2/ERF, NAC, bHLH, and WRKY families). Gradient colors represent the gene expression levels; red represents high expression, whereas green represents low expression.

Transcription regulation is a crucial mechanism when plants encounter with environmental stresses (Wu et al., 2016). In this study, we analyzed the annotations and transcripts of the DEG candidates and detected 362 transcription factors (TFs) distributed across 17 TF families using PlantTFDB (v5.0) (Figure 5A). The dominant TF families were MYB (82 members, 23%), AP2/ERF (56 members, 16%), NAC (43 members, 12%), bHLH (39 members, 11%), and WRKY (29 members, 8%) (Figure 5A). The heatmap showed that the expression patterns of these major TFs were clustered into three groups, and massive TFs were upregulated upon BA stress (Figure 5B).

### Metabolic Response of Peach Plants to Benzoic Acid Treatment

All the BA-induced DEGs were further classified by gene annotation and pathway mapping in the KEGG database. Most of DEGs were distributed into “metabolism” category, including “carbohydrate metabolism,” “biosynthesis of other secondary metabolites,” “amino acid metabolism,” “lipid metabolism,” and “energy metabolism” (Supplementary Figure 2). To further understand the BA-stressed metabolic changes, peach roots treated with 0.4 mM BA for 0 (control), 72, and 120 h were subjected to non-targeted metabolic profiling analysis.

**FIGURE 6:**
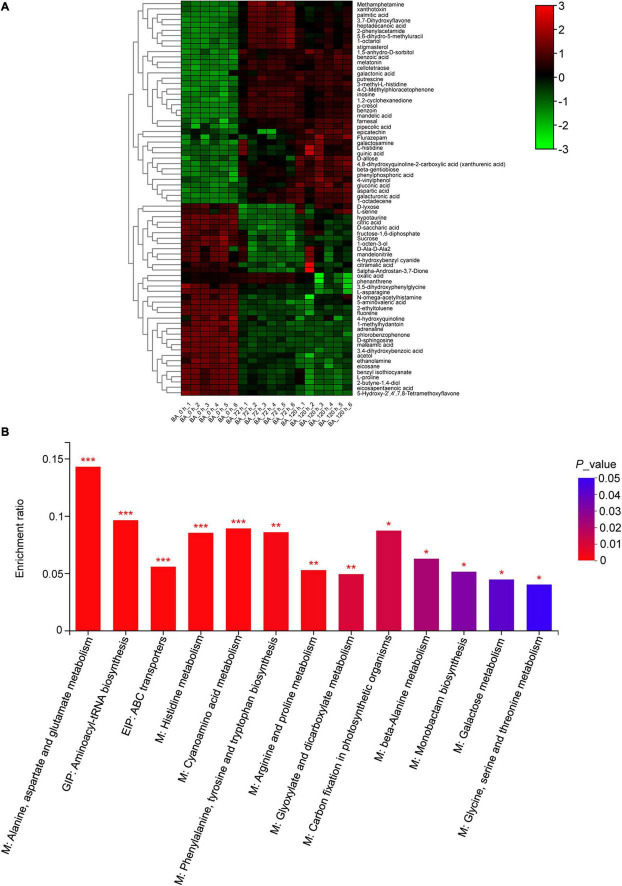
Metabolic changes in the BA-treated peach roots. **(A)** Hierarchical cluster heatmap of differently accumulated metabolites (DAMs) treated with BA for 0, 72, and 120 h. The color changed from green to red, indicating that the abundance of metabolites gradually increases. **(B)** KEGG enrichment results of DAMs. M, metabolism; GIP, genetic information processing; EIP, environmental information processing. The *y*-axis shows the ratio of DAMs to the total background metabolites number in each pathway. The color of the bar changed from blue to red, indicating that the *P-*value gradually decreases. Asterisks on top of bars represent *P-value < 0.05, **P-value < 0.01, and ***P-value < 0.001.

The root metabolite profiling from the BA treated samples (72 and 120 h) was clearly separated from the control along PC1, which explained 47% of the total variance (Supplementary Figure 3). In total, 177 metabolites detected were mainly grouped into primary metabolites, such as sugars, sugar alcohols and derivatives, amino acids and derivatives, organic acids, and fatty acids (Supplementary Table 5). A few secondary metabolites, such as melatonin, putrescine, and polyphenols, were also detected (Supplementary Table 5). Totally, 74 differentially accumulated metabolites (DAMs) were identified, and clustered into five groups based on their abundances under different BA-treated hours (Figure 6A). There were 32 up- and 30 down-accumulated DAMs in 0 h vs. 72 h, and 32 up- and 23 down-accumulated DAMs in 0 h vs. 120 h (Supplementary Figure 4). Furthermore, the KEGG pathway enrichment analysis showed that the 74 DAMs were mainly enriched in 13 pathways, of which, amino acid metabolism related-pathways were the most significantly enriched (Figure 6B).

### Integrative Analysis of Differentially Accumulated Metabolites and Differentially Expressed Genes Under Benzoic Acid Treatment

**FIGURE 7:**
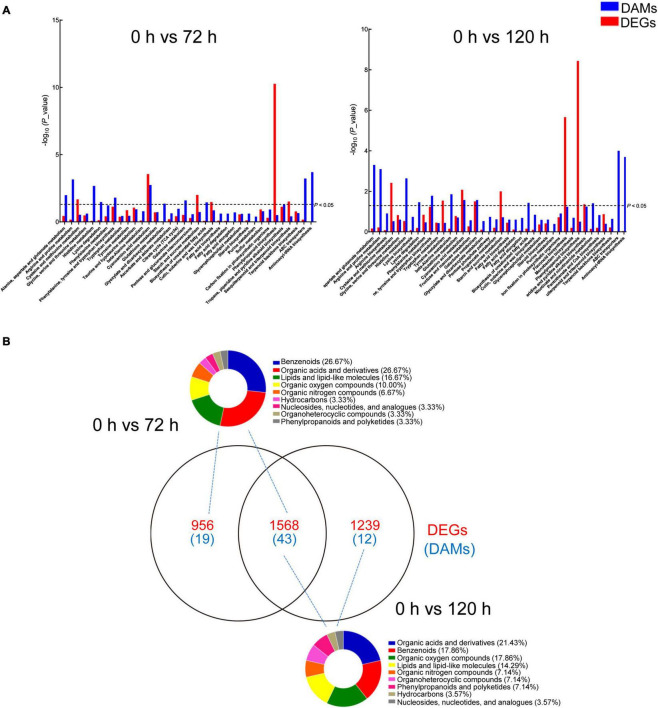
Analysis of DAMs and DEGs in peach roots under BA stress. **(A)** KEGG enrichment analysis of DAMs (blue) and DEGs (red) that are correlated with each other. The *x*-axis represents KEGG pathway subcategories. **(B)** Venn diagram analysis of DEGs (red) and DAMs (blue) in two compared combinations. The DAMs identified from two compared combinations (0 h vs. 72 h and 0 h vs. 120 h) were classified using the Human Metabolome Database (HMDB). The pie graphs represent the classification results, and the different colors represent different compounds categories.

For an in-depth analysis of BA-mediated changes in peach roots, an integrative analysis of transcriptomic and metabolomic data was conducted. A co-joint KEGG enrichment analysis showed that BA caused alterations in the relative amounts of genes and metabolites, which were coincidently grouped into several overlapped pathways. A total of 36 and 40 co-mapped pathways were identified in 0 h vs. 72 h and 0 h vs. 120 h comparisons, respectively. Thirty-one of these pathways were identical after BA treatment at both 72 and 120 h, including “cyanoamino acid metabolism,” “histidine metabolism,” “lysine degradation,” “arginine and proline metabolism,” “alanine, aspartate and glutamate metabolism,” “phenylalanine, tyrosine, and tryptophan biosynthesis,” “glutathione metabolism,” “galactose metabolism,” “starch and sucrose metabolism,” and “phenylpropanoid biosynthesis” (Figure 7A). Most of the co-mapped pathways associated with amino acid and carbohydrate metabolism, indicating that BA stress might affect the nitrogen and carbon metabolism of peach roots. Venn diagram analysis showed the unique/shared DEGs and DAMs sets between two pairwise comparisons (0 h vs. 72 h and 0 h vs. 120 h). The classification results of DAMs based on Human Metabolome Database (HMDB) indicated that most of the DAMs were in categories: organic acids and derivatives, benzenoids, lipids and lipid-like molecules, and organic oxygen/nitrogen compounds, mainly subdivided into amino acids and derivatives, carbohydrates and carbohydrate conjugates, and fatty acids and conjugates (Figure 7B and Supplementary Table 6).

**FIGURE 8:**
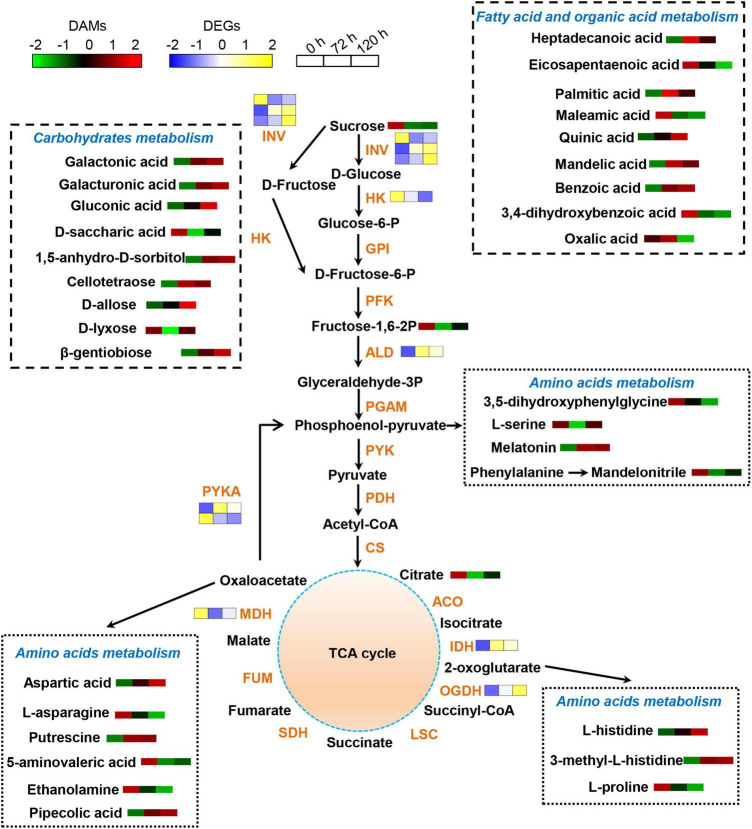
Schematic diagram of proposed metabolic pathways in peach roots in response to BA stress. Pathway was constructed based on the KEGG database and library reference. The abundances of DAMs and DEGs are shown by the heatmap at 0, 72, and 120 h post BA treatment. The color of the heatmap changed from green to red (blue to yellow), indicating that the abundances of DAMs (DEGs) ranging from low to high. INV, invertase; HK, hexokinase; GPI, glucose-6-phosphate isomerase; PFK, 6-phosphofructokinase; ALD, fructose-bisphosphate aldolase; PGAM, 2,3-bisphosphoglycerate-dependent phosphoglycerate mutase; PYK, pyruvate kinase; PDH, pyruvate decarboxylase; CS, citrate synthase; ACO, aconitate hydratase; IDH, isocitrate dehydrogenase; OGDH, 2-oxoglutarate dehydrogenase; LSC, succinyl-coA synthetase; SDH, succinate dehydrogenase; FUM, fumarate hydratase; MDH, malate dehydrogenase; PYKA, phosphoenolpyruvate carboxykinase.

The effects of BA stress on the gene and metabolite network were integrated in a metabolic map (Figure 8). Numerous DAMs were identified to take part in carbohydrate, amino acid, and lipid metabolism. The abundances of sucrose, D-saccharic acid, and D-lyxose were significantly decreased, whereas a significant increase in the accumulation of cellotetraose, 1,5-anhydro-D-sorbitol, galactonic acid, galactonic acid, β-gentiobiose, gluconic acid, and D-allose upon BA exposure (Figure 8). In amino acid metabolism, the abundances of L-serine, ethanolamine, 5-aminovaleric acid, L-asparagine, and L-proline were significantly decreased, whereas the accumulations of L-histidine and aspartic acid were markedly increased. Furthermore, the levels of endogenous BA in roots were gradually increased upon exposure to BA (Figure 8). The BA stress significantly increased the abundances of palmitic acid, mandelic acid, and quinic acid, whereas decreased the abundances of maleamic acid and oxalic acid. In addition, other N-containing compounds, such as pipecolic acid, putrescine, and melatonin were also significantly accumulated in BA-treated roots (Figure 8).

## Discussion

### Benzoic Acid Disturbs Peach Growth by Modulating Physiological and Biochemical Attributes

Peach is a cyanogenic crop in perennial fruit trees, which contains abundant cyanogenic glycosides (Zhu et al., 2017; Shen et al., 2021). Cyanogenic glycosides could be hydrolyzed by β-glycosidase into hydrocyanic acid and BA, which are considered as the major autotoxins in peach replant problem (Zhu et al., 2017). Under autotoxicity stress, plants have equipped with sophisticated responses at physiological, biochemical, transcriptional, and metabolic levels. Plants resort to various strategies to minimize the harmful effects, such as tolerance, resistance, and avoidance (Liang et al., 2019). Therefore, it is necessary to combine multiple approaches to better understand the mechanism of plant response to autotoxicity stress.

In this work, we first investigated plant growth-related parameters under different concentrations of BA treatment. Plant height and fresh biomass of peach seedlings showed a downward trend with increased BA dosage. Root growth was significantly compromised under higher BA concentrations (0.4 and 0.8 mM), which was in agreement with a previous study by Zhu et al. (2017). Allelopathy (or autotoxicity) can induce a set of physiological and biochemical responses in plants (Zhang et al., 2020c). For example, tricin (a principle allelochemical in grain crops) and its related benzothiazine derivatives severely attenuate photosynthetic performance of resistant barnyard grass (Yang et al., 2017). Peach seedlings exhibited a marked decrease in chlorophyll fluorescence (*Fv/Fm*) and chlorophyll content under BA stress. The decreased *Fv*/*Fm* hints that the photosystem II reaction center induces photoinhibition, which will reduce light use efficiency (Jia et al., 2019). Meanwhile, BA significantly decreased the expressions of genes related to light reaction, electron transport chain, chloroplast development, and pigment biosynthesis, such as *STPK*, *CBP*, *CAO*, and *FDX*. Autotoxins trigger the overproduction of ROS, which in turn bring about cytotoxicity (Yang et al., 2018; Zhang et al., 2020c). BA stress induced excess ROS production (H_2_O_2_ and O_2_^•–^) and lipid peroxidation (MDA) in peach roots, especially at higher concentrations. Similar findings have been reported in melon (Zhang et al., 2020c), lettuce (Talukder et al., 2020), and grapevine (Liu et al., 2019a). In consistent with the alterations of ROS levels, the expression levels of many redox-related genes, including *POD*, *CAT*, *SOD*, and *APX* were noticeably downregulated in BA-stressed roots at 120 h. Our results together with previous studies indicate that autotoxicity stress triggers ROS overproduction and lipid peroxidation, thus impairs cellular redox balance, which is a possible reason for BA-mediated growth inhibition (Xie et al., 2017; Yang et al., 2018).

Exposure to autotoxins can also alter mineral uptake and assimilation (Baziramakenga et al., 1994). The BA-treated peach roots showed a significant decrease of P, K, Mg, and Mn contents, which was consistent with a previous work in the BA-treated soybean roots (Baziramakenga et al., 1994). However, Zn was reduced in BA-treated peach roots, but increased in the aforementioned soybean roots (Baziramakenga et al., 1994), which is likely due to the differences in plant species and treatment setup. Moreover, the amount of Fe was increased in roots, but reduced in leaves, which was one of the possibilities for the declined photosynthetic capacity. The uptake and translocation of minerals by plant roots depend on ion transporters, channel proteins, and gated channel proteins (Lai et al., 2020). In this study, BA treatment significantly altered the expression levels of genes coding inorganic ion transporters and channel proteins, which could explain the abnormal mineral nutrition metabolism in stressed peach roots.

### Benzoic Acid Treatment Activates Stress-Responsive Genes

Plants respond to ever-changing environmental challenges via an array of physiological, biochemical, and metabolic modifications, of which activating stress-responsive genes, modulating stress-related substances and their metabolic pathways are common efficient approaches (Wu et al., 2016). In this study, stress-responsive pathways were strongly activated under BA stress. GSTs own versatile roles ranging from plant development, metabolic processes, stress tolerance, to xenobiotic detoxification (Chen et al., 2012). Both GST and GR are important enzymes that regulate cellular reduced glutathione/glutathione disulfide (GSH/GSSG) pool in plant defense mechanisms (Chen et al., 2012). In our study, the expression levels of *GSTs* and *GR* were strongly increased at the early stage of BA treatment, which were in accordance with the results from cinnamic acid-treated melon (Wang et al., 2020). Moreover, exogenous diallyl disulfide (a very important allelochemical in garlic) supply increases GSH content and GST activity that might improve the resistance of tomato roots (Cheng et al., 2016). Our findings together with previous studies suggest that GST and GR may work synergistically to enhance the detoxification of autotoxins.

Benzoic acid noticeably induced the expressions of *sHSPs*, *HSP70*, and *HSP90*, suggesting that HSPs might be closely associated with peach response to BA stress. The roles of HSPs in adjusting plant response to environmental stresses (heat, drought, salt, osmotic, and heavy metal stresses) have been reported in various plant species (Feng et al., 2019; Tian et al., 2021). For instance, *CaHsp25.9* confers pepper plants to heat, salt, and drought tolerance by enhancing the activity of antioxidant enzymes and the expression of stress-related genes (Feng et al., 2019). *MeHSP90.9* could improve cassava drought tolerance by directly regulating abscisic acid and H_2_O_2_ (Wei et al., 2020). Although HSPs have been characterized in many abiotic stresses, their functions in autotoxicity stress remain largely elusive. Therefore, the underlying molecular mechanisms of HSPs involved in the responses to BA stress need future studies.

The transcripts of two genes, *OPR* and *LOX*, which are essentially required for jasmonic acid (JA) biosynthesis (Yin et al., 2020), were significantly induced by BA stress. JA strongly induces allelochemical production at very low concentrations and plays a central role in belowground chemical communications (Kong et al., 2018). To investigate the effect of JA on BA production would provide new insight into reducing BA accumulation in replanted soil (He et al., 2019). ABC transporters, which are a largest family of transporters, involve in the transport of hormones, lipids, and secondary metabolites, as well as detoxification of xenobiotics (Vishwakarma et al., 2019; Zhang et al., 2020c). The expression levels of ABC transporter genes in melon are sharply increased at 24 h under autotoxicity stress (Zhang et al., 2020c). Similarly, a few ABC transporter genes were upregulated in BA stress, implying that ABC transporter might be necessary in the transport and detoxification of autotoxins. Though numerous BA-induced genes were proposed in this work, their precise biological function and regulatory network under BA stress require further exploration.

Plant stress response is also regulated by TFs (Wu et al., 2016; Li et al., 2017). For instance, *PtrNAC72* is a repressor of putrescine biosynthesis and may negatively regulate the drought stress response in trifoliate orange (Wu et al., 2016). The MYB family plays central roles in an array of physiological and biological processes, such as abiotic stress response and metabolic regulation (Li et al., 2017). In the present study, we identified 82 differentially expressed *MYB* genes, and might be involved in peach response to BA stress. In addition to MYBs, a few TFs including AP2/ERF, NAC, bHLH, and WRKY were also potentially associated with BA stress. These results indicated that BA stress altered the expression of many TFs and resulted in transcriptional regulation occurrence. The potential regulatory roles of TFs in BA tolerance ought to be further investigated.

### Benzoic Acid Treatment Redirects Metabolic Programming in Peach Roots

Stressful conditions will inhibit or activate the specific metabolic pathways, and cause stress-specific changes in metabolite levels (Ye et al., 2021). In the present study, BA induced metabolic reprogramming in peach roots. Amino acids play fundamental roles in a wide arrange of plant physiological processes, including ion transport, heavy metals detoxification, redox homeostasis, and enzyme biosynthesis (Zhang et al., 2019; Asaduzzaman and Asao, 2020). Amino acid metabolism was markedly affected by BA stress, indicating that amino acid biosynthesis is a basic response of peach plants to BA stress. Proline is an integral component of cell wall protein formation and an excellent osmotic pressure protective agent (Sharma and Dietz, 2006; Zheng et al., 2020). The contents of proline are remarkably increased in cucumber leaves after being treated with garlic root exudates (Ding et al., 2019). However, BA exposure caused a decrease in the L-proline accumulation, indicating a severe destruction of root cells. Histidine is a heavy metal antidote, and the production of free histidine is closely related to the Ni-hyperaccumulation trait in *Alyssum* species (Sharma and Dietz, 2006). The levels of L-histidine were increased after BA stress, suggesting that it might act as an antidote in alleviating BA poison. In rapeseed leaves, the accumulation of pipecolic acid contributes to osmotic adjustment (Moulin et al., 2006). We found that the contents of pipecolic acid were increased after BA stress, which might be useful for maintaining the osmotic pressure stability of peach roots, thereby reducing BA-mediated damage.

Other N-containing compounds, including putrescine and melatonin were significantly accumulated upon BA stress. Putrescine is a key component of polyamines, and contributes to plant tolerance to diverse stresses by retaining membrane stability, modulating osmotic potential and antioxidant systems (Wu et al., 2016; Li et al., 2017). However, an increase in the endogenous polyamines levels to a certain threshold may promote their degradation, generating H_2_O_2_ (Liu et al., 2015), thus the effects of putrescine on peach seedlings under BA stress need in-depth study in the future. Melatonin is a highly conserved molecule with pleiotropic functions in plant growth, development, and stress adaptation (Li et al., 2018). The elevated levels of putrescine and melatonin suggested their potential roles in peach defense against BA toxicity.

Carbohydrates provide energy for plants adaptation to adverse environments, and serve as a tie in the communication between protein and lipid metabolism (Zhang et al., 2019). The expression levels of genes associated with carbohydrate and amino acid metabolism vary greatly in autotoxic-stressed melon (Zhang et al., 2020c). Similarly, BA changed expression levels of genes involved in carbohydrate and amino acid metabolism. Furthermore, most of carbohydrates including D-allose, cellotetraose, 1,5-anhydro-D-sorbitol, galacturonic acid, galactonic acid, and gluconic acid were significantly accumulated in BA-stressed peach roots. These results indicated that BA partially disturbed carbon metabolism, which might result in energy starvation and growth inhibition. Altogether, the great changes in the amino acid and carbohydrate metabolism indicate that BA stress might affect the nitrogen and carbon pool reallocation in peach roots, which is one of the possibilities for the poor plant growth.

## Conclusion

In summary, BA treatment markedly retarded plant growth with reduced shoot height, root development, and biomass. The BA-stressed plants showed attenuated photosynthetic capacity, as revealed by decreased chlorophyll contents and fluorescence levels, along with downregulated photosynthesis-related genes expression. BA supplementation also gave rise to oxidative stress, reflecting by increased ROS and MDA levels in peach roots. Additionally, an imbalance of mineral nutrient metabolism and a dramatic change of ion metabolism-related genes expression levels were also observed after BA stress. More importantly, numerous differently expressed genes including stress-responsive genes and TFs were identified as being involved in peach response to BA toxicity. A number of differently accumulated metabolites were identified in peach roots, of which amino acid and carbohydrate-related metabolites were dominant. Furthermore, the integrative analysis demonstrated that the carbon and nitrogen metabolism pathways were perturbed in BA-stressed roots. This study provides a valuable insight into the physiological and molecular responsive mechanisms of peach plants to autotoxicity. The information gathered here will also stand as an excellent resource to extensively explore autotoxicity stress resistance genes and metabolites, and assist in plant breeding to alleviate replant problems.

## Data Availability Statement

The datasets presented in this study can be found in online repositories. The names of the repository/repositories and accession number(s) can be found below: https://www.ncbi.nlm.nih.gov/, PRJNA757897.

## Author Contributions

WS, GL, and JL designed the experiments. WS performed the experiments with occasional help from CZ, HZ, and HH. WS, CZ, and WZ analyzed the data. WS, KZ, HZH, and JL wrote the manuscript. All authors read and approved the final manuscript.

## Conflict of Interest

The authors declare that the research was conducted in the absence of any commercial or financial relationships that could be construed as a potential conflict of interest.

## Publisher’s Note

All claims expressed in this article are solely those of the authors and do not necessarily represent those of their affiliated organizations, or those of the publisher, the editors and the reviewers. Any product that may be evaluated in this article, or claim that may be made by its manufacturer, is not guaranteed or endorsed by the publisher.

## Footnotes

1
http://www.graphpad.com

2
https://cloud.majorbio.com
