# Supplementary material for: Integrative Physiological, Transcriptional, and Metabolic Analyses Provide Insights Into Response Mechanisms of Prunus persica to Autotoxicity Stress
Source: Front Plant Sci. 2021 Dec 15;12:794881. doi: 10.3389/fpls.2021.794881 (PMC8714634; doi:10.3389/fpls.2021.794881)
Supplement: Supplementary file 1 [file Presentation_1.PPTX]

## Slide 1
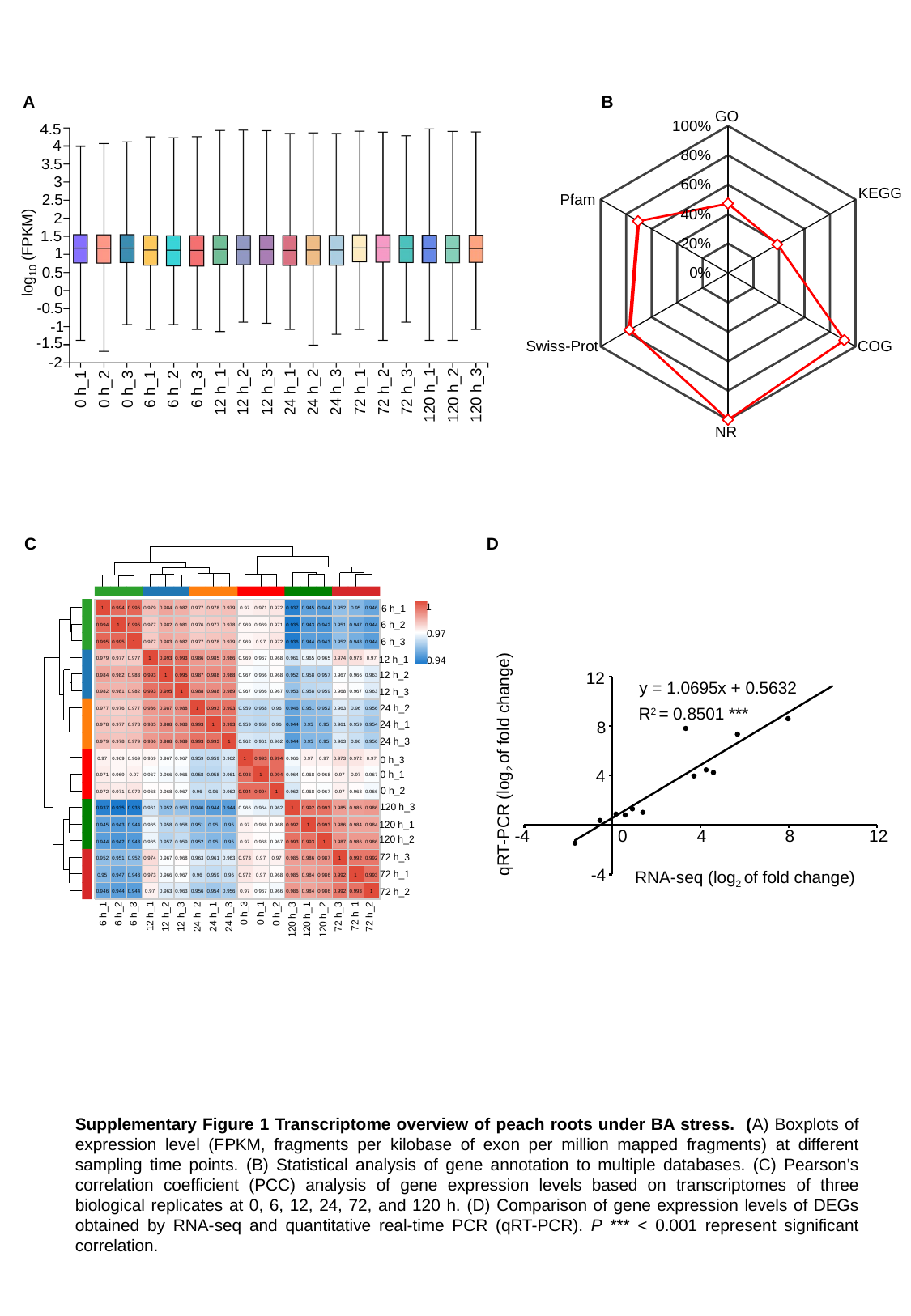

A
B
C
D
12
y = 1.0695x + 0.5632
R2 = 0.8501 ***
8
qRT-PCR (log2 of fold change)
4
-4 0 4 8 12
-4
RNA-seq (log2 of fold change)
Supplementary Figure 1 Transcriptome overview of peach roots under BA stress. (A) Boxplots of expression level (FPKM, fragments per kilobase of exon per million mapped fragments) at different sampling time points. (B) Statistical analysis of gene annotation to multiple databases. (C) Pearson’s correlation coefficient (PCC) analysis of gene expression levels based on transcriptomes of three biological replicates at 0, 6, 12, 24, 72, and 120 h. (D) Comparison of gene expression levels of DEGs obtained by RNA-seq and quantitative real-time PCR (qRT-PCR). P *** < 0.001 represent significant correlation.

## Slide 2
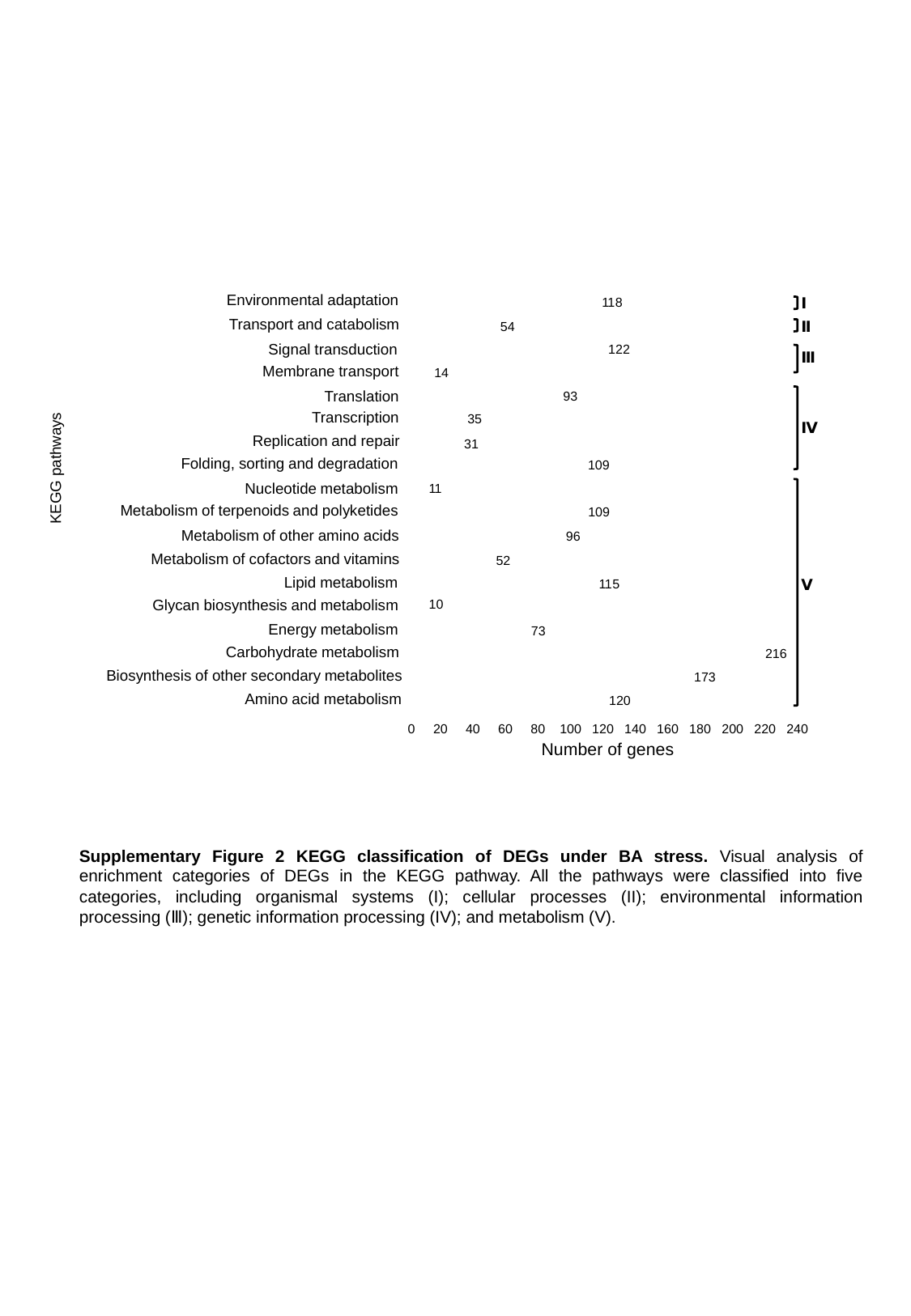

Supplementary Figure 2 KEGG classification of DEGs under BA stress. Visual analysis of enrichment categories of DEGs in the KEGG pathway. All the pathways were classified into five categories, including organismal systems (I); cellular processes (II); environmental information processing (Ⅲ); genetic information processing (IV); and metabolism (V).

## Slide 3
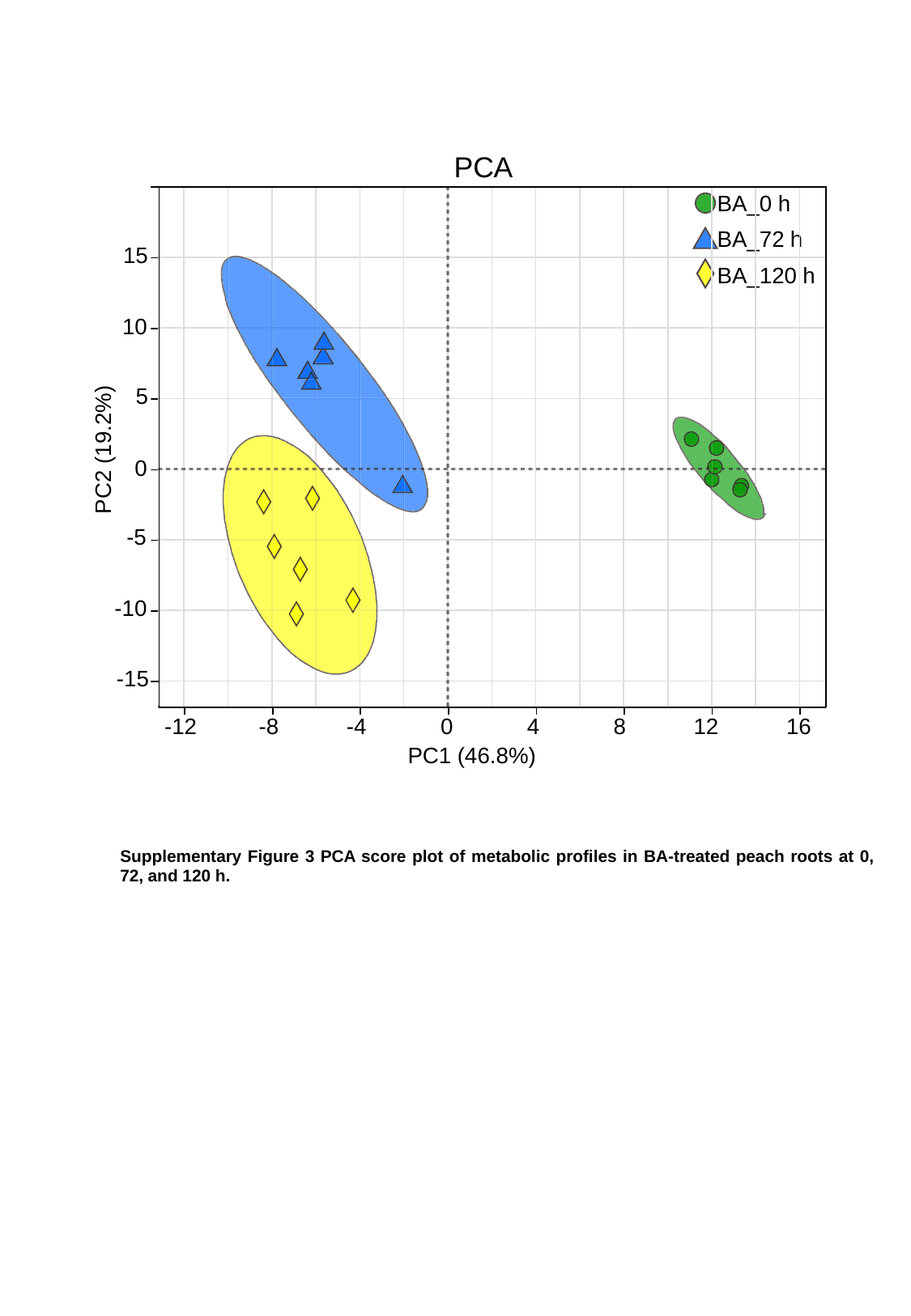

Supplementary Figure 3 PCA score plot of metabolic profiles in BA-treated peach roots at 0, 72, and 120 h.

## Slide 4
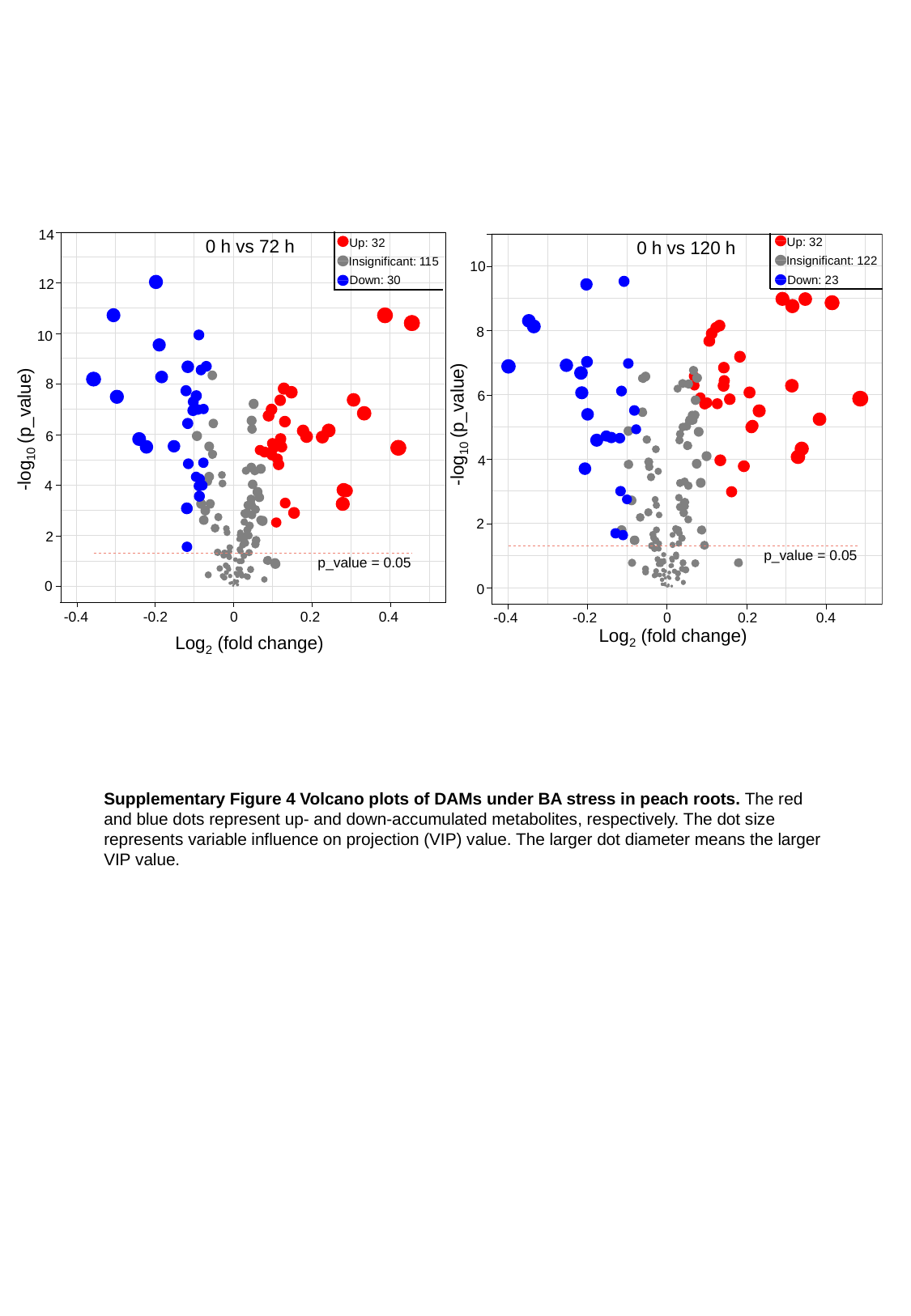

Supplementary Figure 4 Volcano plots of DAMs under BA stress in peach roots. The red and blue dots represent up- and down-accumulated metabolites, respectively. The dot size represents variable influence on projection (VIP) value. The larger dot diameter means the larger VIP value.
